# Comprehensive EST analysis of the symbiotic sea anemone, *Anemonia viridis*

**DOI:** 10.1186/1471-2164-10-333

**Published:** 2009-07-23

**Authors:** Cécile Sabourault, Philippe Ganot, Emeline Deleury, Denis Allemand, Paola Furla

**Affiliations:** 1 Université de Nice-Sophia-Antipolis, EA4228 ECOMERS, Nice, France; 2 Centre National de la Recherche Scientifique, UMR6243, Sophia-Antipolis, France; 3 Université de Nice-Sophia-Antipolis, UMR6243, Sophia-Antipolis, France; 4 Institut National de la Recherche Agronomique, UMR6243, Sophia-Antipolis, France; 5 Centre Scientifique de Monaco, Principauté de Monaco

## Abstract

**Background:**

Coral reef ecosystems are renowned for their diversity and beauty. Their immense ecological success is due to a symbiotic association between cnidarian hosts and unicellular dinoflagellate algae, known as zooxanthellae. These algae are photosynthetic and the cnidarian-zooxanthellae association is based on nutritional exchanges. Maintenance of such an intimate cellular partnership involves many crosstalks between the partners. To better characterize symbiotic relationships between a cnidarian host and its dinoflagellate symbionts, we conducted a large-scale EST study on a symbiotic sea anemone, *Anemonia viridis*, in which the two tissue layers (epiderm and gastroderm) can be easily separated.

**Results:**

A single cDNA library was constructed from symbiotic tissue of sea anemones *A. viridis *in various environmental conditions (both normal and stressed). We generated 39,939 high quality ESTs, which were assembled into 14,504 unique sequences (UniSeqs). Sequences were analysed and sorted according to their putative origin (animal, algal or bacterial). We identified many new repeated elements in the 3'UTR of most animal genes, suggesting that these elements potentially have a biological role, especially with respect to gene expression regulation. We identified genes of animal origin that have no homolog in the non-symbiotic starlet sea anemone *Nematostella vectensis *genome, but in other symbiotic cnidarians, and may therefore be involved in the symbiosis relationship in *A. viridis*. Comparison of protein domain occurrence in *A. viridis *with that in *N. vectensis *demonstrated an increase in abundance of some molecular functions, such as protein binding or antioxidant activity, suggesting that these functions are essential for the symbiotic state and may be specific adaptations.

**Conclusion:**

This large dataset of sequences provides a valuable resource for future studies on symbiotic interactions in Cnidaria. The comparison with the closest available genome, the sea anemone *N. vectensis*, as well as with EST datasets from other symbiotic cnidarians provided a set of candidate genes involved in symbiosis-related molecular crosstalks. Altogether, these results provide new molecular insights that could be used as a starting-point for further functional genomics studies.

## Background

Sea anemones, together with corals, jellyfish and hydras, belong to the Cnidaria, which are basal to the eumetazoa and ancestral to the bilateria. Cnidaria are characterized by a sac-like body plan with a single oral opening surrounded by numerous tentacles. As diploblastic animals, they are composed of only two embryonic tissue layers, the epiderm and the gastroderm (Additional file 1).

Many cnidarians harbour photosynthetically active unicellular algae within their gastrodermal cells. In most cases, such symbiont algae are dinoflagellates from the genus *Symbiodinium*, commonly referred to as zooxanthellae. This association is a trophic endosymbiosis and is considered to be mutualistic because the zooxanthellae provide their cnidarian host with reduced organic carbon resulting from their photosynthetic activity [1] while the host provides the zooxanthellae with inorganic carbon [2], inorganic nitrogen [3,4] and inorganic phosphate [5], as well as a refuge from herbivory. This simple mutual partnership has recently been revealed to be more complex, however, since the holobiont was found to be a dynamic assemblage of animal, zooxanthellae, endolithic algae and fungi, prokaryotes (Bacteria and Archaea) and viruses [6,7]. Endosymbioses are thus highly complex associations, implying intimate interactions between host and symbionts as well as constraints, such as hyperoxic conditions generated by symbiont photosynthesis, and transfer of inorganic carbon to the symbiont [8].

In recent decades, biochemical and physiological studies have highlighted numerous adaptations in cnidarian host tissues (for review [8]), such as the presence of natural sunscreens (UV-absorbing mycosporine-like amino acids, [9]), remarkable antioxidant defences [10,11], specific mechanisms of inorganic carbon absorption and concentration [12], and mechanisms of inorganic nitrogen absorption [3]. However, despite increasing knowledge about their physiological inter-relationship, very little is known about the molecular adaptations that have permitted this successful partnership.

The cnidarian-dinoflagellate endosymbiotic association is the very foundation of the highly productive and diversified coral reef ecosystem. Coral reefs are considered to host at least 30% of all known marine fauna [13], like "oases" within marine nutrient-deprived deserts [14], and play a crucial role in shaping tropical ecosystems. Coral reefs are, however, now also experiencing high levels of anthropogenically-induced stress (global climate change, pollution). Such environmental perturbations, in addition to pathogens, contribute to the breakdown of symbiosis known as "coral bleaching", and even mortality [15]. Bleaching results in whitening of cnidarian symbiotic tissues, due either to a direct loss of dinoflagellates and/or a decrease in photosynthetic pigment concentration [16]. Mass bleaching events have been increasing in both frequency and severity since the 1980s [15].

The most significant contributions to cnidarian molecular biology are the complete genome analysis of the starlet Sea anemone *Nematostella vectensis *[17-19] and the *Hydra magnipapillata *genome project [20]. However, neither of these species is symbiotic. In addition, phylogenetic studies suggest that *N. vectensis *and *A. viridis *might belong to different suborders (Additional file 2), although both belong to the Hexacorallia Actiniaria [21]. Very few studies using high-throughput techniques have been published to date on symbiotic cnidarians. Most cDNA libraries have been constructed from non-symbiotic cells and only limited EST datasets were generated. Only three studies have been conducted on symbiotic anthozoans: *Acropora millepora*, a reef building coral (10,247 ESTs; [19,22]); *Aiptasia pulchella*, a tropical Sea anemone (870 ESTs; [23]); and a comparative study on two scleractinian corals, *Montastrea faveolata *(3,854 ESTs) and *Acropora palmata *(14,588 ESTs, [24]). All have shown that cnidarians are more similar to vertebrates, in terms of gene repertoire, composition and intron/exon structure, than the well known ecdysozoan model organisms, *Drosophila melanogaster *and *Caenorhabditis elegans*, where extensive gene loss has occurred [19,22]. These EST datasets have been used to develop small-scale microarrays for gene expression analysis in several coral-algal symbioses: *Acropora millepora*, *Acropora palmata *and *Montastrea faveolata *[25-27].

Genomic information on zooxanthellae has been obtained by Leggat et al [28] who analysed 2,682 ESTs of tropical *Symbiodinium *(clade C3) extracted from the coral *Acropora aspera*. Another cDNA library (1,484 UniSeqs) was constructed from a cultured *Symbiodinium *of clade A (CassKB8), originally isolated from the Upside-down jellyfish *Cassiopea *sp, and sequences were compared to those of clade C3 [29]. Additional metagenomic analyses were performed on the microbial community associated with the coral *Porites asteroids *[7,30], extending our knowledge on the organismal diversity of the holobiont, although no transcriptomic analyses were carried out.

To better characterize symbiotic relationships between cnidarian host and associated symbionts, we conducted a large-scale EST study on a symbiotic sea anemone. We chose the sea anemone *Anemonia viridis *as our study species, in which the two tissue layers (epiderm and gastroderm) can be easily separated. *A. viridis *is the most abundant sea anemone of the Mediterranean coasts and hosts the temperate *Symbiodinium *sp. of Clade A, which has been suggested to be the dominant clade of zooxanthellae in the Mediterranean Sea [31]. We prepared a single cDNA library from whole specimens under several stress conditions, in order to maximize the presence of genes required for symbiosis. 39, 939 ESTs were generated and a total of 14,504 UniSeqs were identified, assigned a putative origin and annotated. This large collection of UniSeqs provides the better characterized transcriptomic knowledge of symbiosis in cnidarians. The data will further our comprehension of such relationships and contribute to functional genomic surveys.

## Results and discussion

### ESTs generation and analysis

**Figure 1:**
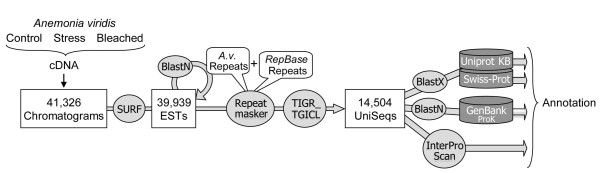
**Flowchart of the analysis pipeline of *A. viridis *ESTs**. EST processing and analysis pipeline used in this study.

A cDNA library was made from both symbiotic (gastroderm) and non-symbiotic (epiderm) tissues of the sea anemone *Anemonia viridis *(Additional file 1). In order to maximize the diversity of genes expressed under both normal and stress conditions within the symbiotic association, sea anemones were subjected to different environmental conditions before RNA extraction and cDNA library construction (light/dark sampling, thermal stress, hyperoxia conditions, see Materials & Methods). Out of 50,304 sequenced clones, a total of 41,247 readable sequences were produced, corresponding to a sequencing success of 82%, and 39,939 high quality ESTs were generated (Figure 1 and Additional file 3). *A. viridis *repetitive sequences were identified and masked using RepeatMasker before assembly. Trimmed masked ESTs were further subjected to cluster analysis using the TIGR-TGICL pipeline with default parameters [32]. A total of 30,087 ESTs were assembled into 4,652 contigs (Additional file 4) while 9,852 sequences remained as singletons. The mean trimmed EST sequence length was 626 bp, but sequences could be assembled into contigs of up to 4 kb (CL4Contig4, described as ribosomal protein L8, 3,887 bp). Most contigs (2,959) were composed of three ESTs or less, with an average length of 895 bp, suggesting that they covered only parts of the transcript sequences. Apart from the functional genomics resources available for the reef-building corals *Acropora palmata *and *Montastrea faveolata *[24], most cnidarian genomic studies have been performed on non-symbiotic tissues (aposymbiotic developmental stages or adults). To our knowledge, our study provides the largest EST collection from a symbiotic cnidarian.

The most abundant transcripts (contigs composed of more than 100 ESTs) are listed in Table 1. Most of these correspond to ribosomal proteins, or proteins involved in basic cellular processes (actin, elongation factor 1 alpha, CAAT enhancer binding protein). However, it should be highlighted that two of these highly abundant transcripts had no similarity with any available sequence, either in UniProt or NR databases, while two other large clusters represent proteins that might be involved in stress responses (HSP70 and ferritin). Ferritins are ubiquitously expressed proteins that have a central role in normal iron homoeostasis, as well as during oxidant stress, by reducing the participation of iron in free-radical-generating reactions [33]. Ferritin genes have also been identified as being highly expressed in *Acropora *EST datasets and one has been shown to be under positive selection [24]. Microarray experiments, designed to identify genes differentially expressed in response to elevated temperature in *Anthopleura elegantissima *[34] or *A. viridis *(data not shown), revealed that ferritin genes might be induced in response to increases in reactive oxygen species following thermal stress.

**Table 1:** The 19 most abundant transcripts (>100 ESTs)

					**Top hit Swissprot (2008.03)**			
					
**Cluster ID**	**Total ESTs**	**Length (bp)**	**Description**	***N. vectensis *top hit** **(Uniprot ID)**	**Accession**	**Name**	**Organism**	**E-value**
CL1Contig11	828	478	CCAAT/enhancer-binding protein beta	A7S4U3_NEMVE	O02755	CEBPB_BOVIN	*Bos taurus*	5E-17
CL1Contig8	482	1892	Elongation factor 1-alpha	A7SSW8_NEMVE	P19039	EF1A_APIME	*Apis mellifera*	0
CL2Contig7	340	1411	Actin, cytoplasmic	A7SCN8_NEMVE	Q964E3	ACTC_BIOAL	*Biomphalaria alexandrina*	0
CL3Contig4	334	764	Soma ferritin	A7S8I6_NEMVE	P42577	FRIS_LYMST	*Lymnaea stagnalis*	4E-68
CL1Contig4	322	968	lipoprotein PA4545 precursor	*No hits found*	P33641	Y9F5_PSEAE	*Pseudomonas fluorescens*	2E-31
CL4Contig4	311	3887	60S ribosomal protein L8	A7S5T9_NEMVE	Q9U9L2	RL8_ANOGA	*Anopheles gambiae*	2E-35
CL5Contig1	234	855	*No hits found*	*No hits found*				
CL11Contig1	175	2915	Translation elongation factor 2	A7RSB9_NEMVE	Q1HPK6	EF2_BOMMO	*Bombyx mori*	0
CL3Contig3	172	1418	Pancreatic secretory granule membrane major glycoprotein GP2	A7S5P2_NEMVE	P25291	GP2_CANFA	*Canis lupus familiaris*	2E-18
CL12Contig1	170	2227	Polyadenylate-binding protein 4	A7SSV8_NEMVE	Q13310	PABP4_HUMAN	*Homo sapiens*	0
CL13Contig1	152	887	40S ribosomal protein S2	A7RJJ7_NEMVE	P49154	RS2_URECA	*Urechis caupo*	1E-114
CL2Contig1	148	1487	Actin, cytoskeletal 1A	A7RU31_NEMVE	P53472	ACTA_STRPU	*Strongylocentrotus purpuratus*	0
CL7Contig2	145	1224	ADP, ATP carrier protein, mitochondrial precursor	A7RN38_NEMVE	P31691	ADT_ORYSJ	*Oryza sativa*	1E-104
CL1Contig3	138	528	*No hits found*	*No hits found*				
CL14Contig1	134	1084	60S acidic ribosomal protein P0	A7SQ36_NEMVE	P47826	RLA0_CHICK	*Gallus gallus*	1E-108
CL18Contig1	108	1100	40S ribosomal protein SA	A7RKS5_NEMVE	P50890	RSSA_CHICK	*Gallus gallus*	3E-94
CL20Contig1	106	2872	40S ribosomal protein S3a	A7S3J7_NEMVE	P49242	RS3A_RAT	*Rattus norvegicus*	1E-100
CL19Contig1	105	847	Heat shock 70 kDa protein cognate 4	A7SG65_NEMVE	Q9U639	HSP7D_MANSE	*Manduca sexta*	0
CL6Contig3	102	866	40S ribosomal protein S4	A7SRV8_NEMVE	P47961	RS4_CRIGR	*Cricetulus griseus*	1E-112

### Repeated elements

Based on reverse transcriptase domain search, 46 transposable elements (retrotransposons/retroposons) were identified (data not shown). Among these, 4 were of prokaryotic origin (without any similarity to *N. vectensis *sequences) and 42 were of metazoan origin. While 2 of them were almost identical to *N. vectensis *transposable elements, 21 only had slight similarity to *N. vectensis *sequences (BlastX or BlastN with E-value of 1.10^-25 ^to 1.10^-10^), and 19 showed no similarity to *N. vectensis *sequences but had been previously identified in other Metazoa.

**Figure 2:**
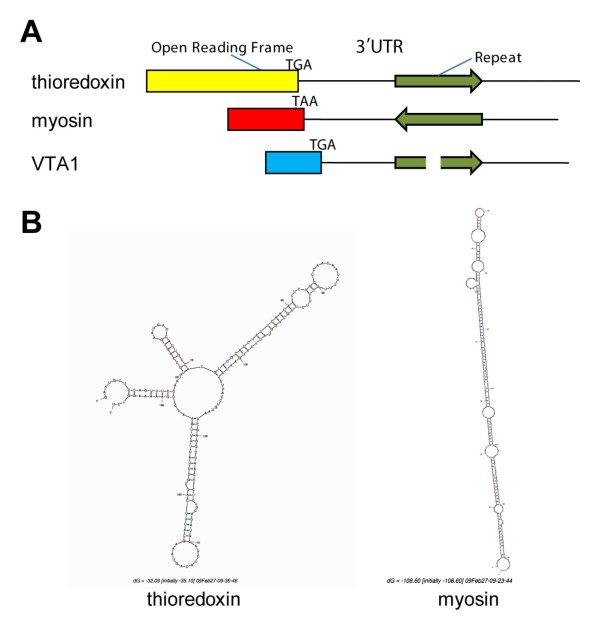
**Representative repeated element found in many 3'UTRs of *A. viridis *coding genes**. A: Schematic representation of the same repeated element found downstream of the ORF of the following *A. viridis *homologs: thioredoxin, myosin (opposite orientation) and VTA1 (truncated form). B: Calculated secondary structure (Mfold2.3 program) of the two first repeats, showing the formation of an extremely stable hairpin in the 3'UTRs of the myosin gene.

Self BlastN analysis on our EST dataset also identified another abundant set of repeated elements. Over 1,500 repetitive sequences were identified, with 412 repeated more than 10 times in our UniSeqs dataset. A typical sequence would be around 200 bp length, harbouring 40–50 bp inverted terminal repeats at its extremities. These repeats often formed very long and stable hairpins, depending on their orientation (Figure 2). They were found in 19.8% of UniSeqs and were overrepresented within contigs (62% of contigs contained at least one repeat). When open reading frame could be identified, these *A. viridis *repeated elements were always mapped in the 3'UTRs of the protein coding genes. Using a BlastN analysis, none of these sequences were found either in the genome of *N. vectensis *or in the large EST datasets of *Hydra magnipapillata*, and search in Repbase did not show any homologs. However, members of these *A. viridis *repeated elements could be found in the limited EST datasets available for the symbiotic coral *Acropora millepora *and the sea anemone *Anthopleura elegantissima*. Further structural and functional characterisations of these repeated elements are currently being made.

### Comparative analysis to related taxa

**Figure 3:**
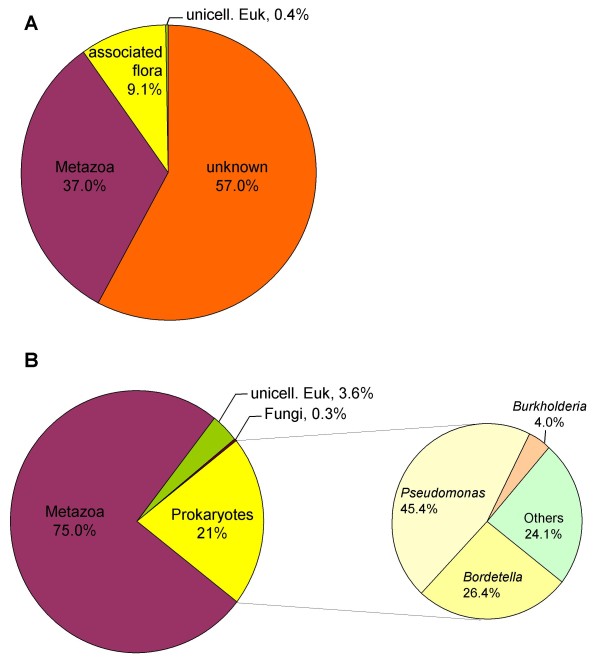
**Distribution of BLAST results**. Pie charts of the distribution of UniSeqs by organism. A: Distribution of all UniSeqs (unknown vs annotated sequences). B: Distribution of annotated UniSeqs. The sequences were annotated using the pipeline shown in Figure 3, and assigned a putative origin based on the Blast results. The term "putative associated flora" covers sequences from prokaryotes (21% of annotated sequences), fungi (0.3%) and viruses (0.1%).

**Figure 4:**
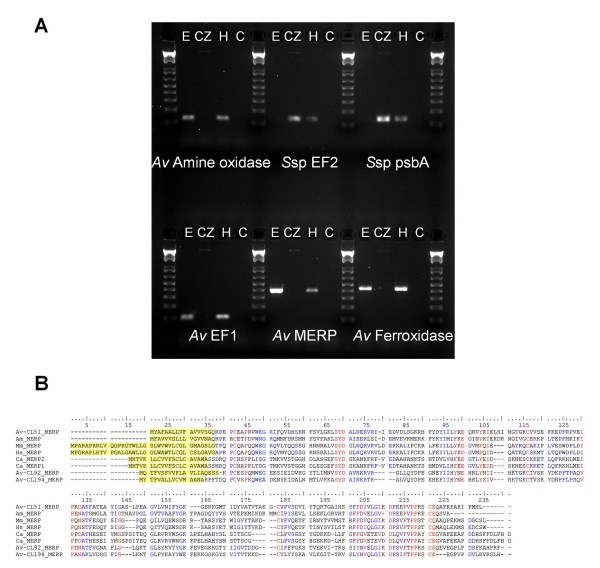
**Evidence for specific metazoan genes isolated from *Anemonia*, but not found in the *Nematostella *genome**. A: PCR results confirming the metazoan origin of MERP, Ferroxidase and Amine-oxidase genes. Amplifications were performed on genomic DNA from ectodermal cells (specific to *A. viridis*, **E**), cultured zooxanthellae (specific to *Symbiodinium*, **CZ**), or holobiont tissues (both *A. viridis *and *Symbiodinium*, **H**), and water as a negative control (**C**). Three other specific genes were used to confirm the resulting profiles: *S*sp *EF2 *is the nuclear gene for *Symbiodinium *sp elongation factor 2, *S*sp *psbA *is the chloroplast-encoded gene for *Symbiodinium *sp photosystem II protein D1, and *Av EF1 *is the nuclear gene for *A. viridis *elongation factor 1 alpha. B: Amino acids alignment of several MERP sequences from *Anemonia viridis *(Av) *Acropora millepora *(Am), *Mus musculus *(Mm), *Homo sapiens *(Hs) and *Carassius auratus *(Ca). Signal peptides are highlighted in yellow.

Because the cDNA library was made from symbiotic tissue, we expected to find ESTs related both to the cnidarian host and to its dinoflagellate symbionts. The analysis pipeline used in this study is presented in Figure 1. Sequences were compared with SwissProt (2008.03) and Uniprot KB (TrEMBL+Swissprot) (2008.03) databases using BlastX with a cutoff E-value of < 1.10^-10 ^to retrieve functional annotations. Of the assembled dataset, 6,238 UniSeqs had putative similarities while 8,266 had no similarity to any sequences in the chosen databases. Sequences were also compared with NCBI-indexed prokaryotic nucleic sequences (2007.08 release), using BlastN with a stringent E-value of < 1.10^-15 ^to assess the proportion of prokaryotic sequences. Finally, a specific search of the UniSeqs for virus proteins returned 7 hits. Relative contribution is shown in Figure 3. First of all, a relatively high proportion of sequences (57%) remained that showed no significant similarities to previously described genes and were therefore considered as 'unknown'. This is somewhat comparable with results obtained from other cnidaria, *Acropora palmata*, *Acropora millepora*, *Montastrea faveolata*, and *Nematostella vectensis *[19,24]. Most of the UniSeqs identified in *Symbiodinium *sp were also of unknown origin [28]. Metazoan hits were found for 32.3% of UniSeqs (75% of annotated sequences). Among these, most of the annotated UniSeqs (4,266 out of 4685) matched with *Nematostella vectensis *predicted proteins. However, we also identified sequences that were clearly from the host (first Blast hits of metazoan origin with cutoff E-value < 1.10^-50^), but these had no significant similarity to predicted proteins of *N. vectensis*. Three of these (a glycoprotein, a ferroxidase and an amine-oxidase) were studied in more detail (Figure 4). PCR and sequencing were first performed on genomic DNA from both *A. viridis *epiderm and *in vitro *cultured *Symbiodinium*, which confirmed the animal origin of these sequences (Figure 4A). The first sequence studied is related to the ependymin glycoprotein family (more specifically to the Mammalian Ependymin-Related Proteins group or MERP1), which has been well described in vertebrates due to its involvement in the regeneration processes. Ependymins are secretory proteins that can bind calcium and that were found predominantly in the cerebrospinal fluid of teleost fish. A bound form has been described, associated with the extracellular matrix. Recent data demonstrated that these proteins are also present in non-vertebrate deuterostomes and protostomes [35], and that positive selection may have shaped their evolution. Figure 4B illustrates an amino acid alignment of *A. viridis *MERP sequences to homologous proteins found in publicly available databases (Bayesian analysis of the MERP sequence confirmed the phylogenetic relationship, not shown). The presence of ependymin proteins in basal organisms clearly indicates for the first time that this protein family is far older than previously thought (first described as chordate-specific, then deuterostome-specific, and finally also found in protostomes).

In addition, there are at least 3 distinct MERP homologues in *A. viridis*, all of animal origin. As this is never the case in other phyla (only one homologue was found in any other species), we could hypothesize that MERP are important for symbiosis in cnidarians. The second sequence is related to a copper-dependent ferroxidase family protein (hephaestin) involved in copper detoxification, which has been described both in eukaryotes (unicellular eukaryotes and vertebrates) and prokaryotes. Nevertheless, no significant similarity was found in *D. melanogaster *or in *C. elegans *genomes, nor in any available protostome database, suggesting that this has been lost during protostome evolution. The third sequence is related to a prokaryotic amine-oxidase (permease) family protein, which has also been identified in the genome of *C. elegans *and other Caenorhabditidae but not in any other animal sequence database. Such species-specific occurrence could be explained by the presence of a similar prokaryote in both nematoda and *A. viridis *associated flora (sequence contamination) or a parallel gene transfer event during their evolution. However, molecular phylogenetic analyses (neighbor joining, maximum parsimony and maximum likelihood) showed that cnidarian and rhabditidae nematode sequences cluster to outside other prokaryote representative gene sequences (Additional file 5). The latter rather suggest that amine-oxidase genes were maintained in these taxa but lost in other metazoa.

Quite a large proportion of UniSeqs (9%) were uniquely shared with prokaryotes, of which *Proteobacteria *was the most prominent bacterial group (*Pseudomonas*, *Bordetella*, and *Burkholderia*). The holobiont has already been described as a dynamic assemblage, made up of the animal host, zooxanthellae, endolithic algae and fungi, Bacteria, and Archea [7]. Such "prokaryotic sequences" could therefore be assigned to the sea anemone-associated flora. In addition, a small but significant number of *A. viridis *sequences, recognized as being of prokaryotic origin based on Blast analysis, had already been identified in the *N. vectensis *genome. Such genes are clearly similar in sequence to prokaryotic homologs, although they contain introns. In *N. vectensis *and *A. millepora *they were proposed as "ancient genes", conserved in cnidarians but lost in other animal genomes [19]. Although our results are in line with this interpretation, comparative genomic studies among cnidarians, such as *A. viridis*, could help to identify the most probable evolutionary scenario between maintenance of "non-metazoan" genes in cnidarians or lateral gene transfer events, followed by rapid intron acquisition.

Surprisingly, only a small fraction of our dataset could be assigned to unicellular eukaryote sequences (putative *Symbiodinium *sp, 3.6% of annotated sequences). Two well accepted explanations have been proposed: i) poor representation of dinoflagellate sequences in databases, leading to wrong assignment after Blast analysis; and ii) technical bias due to the *Symbiodinium *cell wall impairing complete RNA extraction with standard methods, thus leading to an under-representative number of cDNAs in our library.

**Figure 5:**
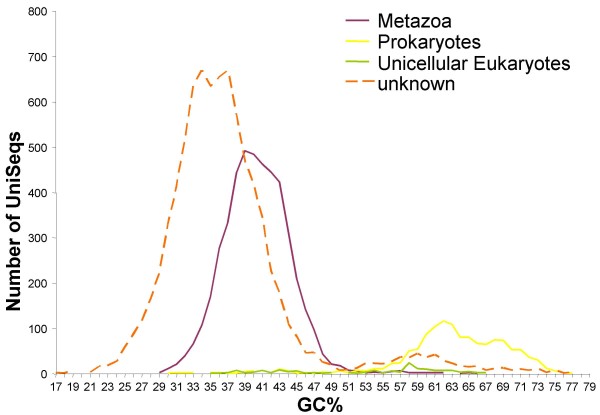
**GC-content distribution of unique sequences**. The GC-content was calculated for all UniSeqs and compared with Blast results to assign a putative origin to the sequences. The GC-content distribution is presented by putative origin for annotated sequences (*A. viridis*, *Symbiodinium *or associated flora). Results related to sequences of unknown function (dashed line) are discussed in the text.

GC content was calculated for all UniSeqs to better assign a source to the transcripts (species of origin). Figure 5 represents the distribution of GC content in the three major taxa (metazoans, unicellular eukaryotes and associated prokaryotic flora), as well as for sequences without BlastX hits (unknown). Two distinct distribution curves were observed, with the metazoan ESTs averaging 40% GC, while bacterial ESTs averaged 63%. Despite our small number of *Symbiodinium *sequences, their GC content (58%) agreed with that already published for dinoflagellates, which have a relatively high GC content compared with other eukaryotes: Triplett et al [36] calculated a GC content of 64% for *Heterocapsa pygmaea *and an EST analysis of *Alexandrium tamarense *estimated that coding region GC-content was 60.8%, whereas GC-content in the 3'-UTR was slightly less, at 57.6% [37]. GC-content analysis of our unknown sequences revealed that ESTs were clustered around two peaks corresponding to 35% and 58% GC content for the larger and smaller peaks respectively. These data strongly suggest that most of the unknown sequences are likely to be of animal origin. As the GC content of 3' UTRs is usually lower than that of 5' UTRs or CDSs, these results are also consistent with our hypothesis that these sequences are mainly 3' UTR (due to cloning strategy), which also explains their low level of annotation. On the other hand, the smaller peak (58% GC) could be attributed to dinoflagellate sequences, although no dinoflagellate genome is currently available to test this, and the most closely related available genomes are those of apicomplexans (*Plasmodium falciparum *and *Toxoplasma gondii*), which are also compact genomes of parasitic protozoans.

**Figure 6:**
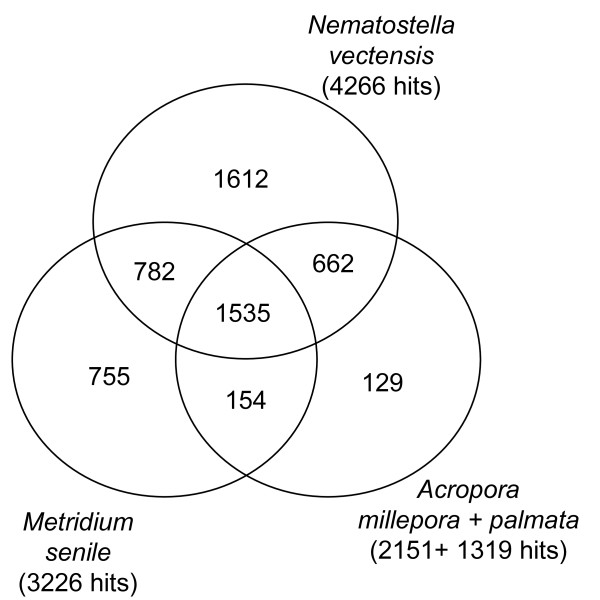
**Venn diagram illustrating the distribution of positive blast hits between *A. viridis *and selected Cnidaria**. Positive hits were identified using tBlastX (ESTs from *M. senile *and *Acropora*) or BlastX (predicted proteins dataset of *N. vectensis*), using a cutoff E-value of < 1.10^-10^. 129 genes are common to the symbiotic species, *A. viridis *and at least one species of *Acropora*.

To gain some insight into genes potentially involved in symbiosis among cnidaria, we performed comparative Blast analyses on the subpart of *A. viridis *sequences that were of animal origin (12,448 sequences). They were subjected to tBlastX (cutoff E-value of < 1.10^-10^) against cnidarian EST sequences available in NCBI-dbEST (2009.05). A parallel BlastX (cutoff E-value of < 1.10^-10^) was performed against the *N. vectensis *predicted proteins dataset. Table 2 gives the number of positive Blast hits (presence vs absence) obtained from *A. viridis *sequences against ESTs from selected cnidarian species. This comparison highlighted two species with a high number of homologous sequences, the sea anemone *M. senile *and the symbiotic stony corals *A. millepora *and *A. palmata*. In the Figure 6 Venn diagram we compared *A. viridis *with two non symbiotic species (*N. vectensis *and *M. senile*), and the symbiotic *Acropora *species: 1,535 sequences were common to the four species, while only 129 sequences (of which 88 have no BlastX hit) were specific to the symbiotic species, which probably contain candidate genes required for the symbiosis.

**Table 2:** Distribution of positive blast hits among Cnidaria

	tblastx (1e-10)	
	
Species	Number of ESTs	Number of hits
*Hydra magnipapillata*	164325	2355
*Nematostella vectensis*	163314	4475
*Metridium senile*	29412	3226
*Clytia hemisphaerica*	27674	1637
*Hydra vulgaris*	18830	992
*Acropora palmata*	13945	1319
*Porites astreoides*	11516	1306
*Aiptasia pallida*	10285	1648
*Acropora millepora*	10247	2151
*Hydractinia echinata*	9460	951
*Montastraea faveolata*	3873	487
	blastx (1e-10)	
	
*Nematostella vectensis*	27273^1^	4266

Sequences identified as metazoan origin (12,448 sequences) were compared to cnidarian sequences available in databases.

^1^predicted proteins dataset from JGI

### Functional classification of ESTs

**Figure 7:**
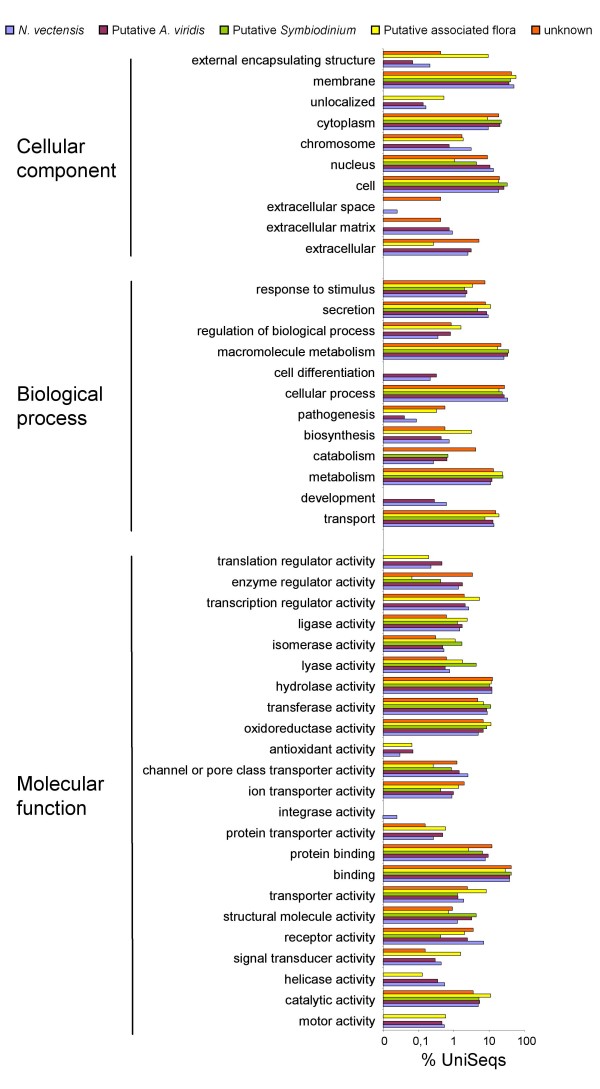
**Distribution of gene ontology terms**. From the *A. viridis *library, a total of 29% of the UniSeqs were assigned GO terms. Unique sequences were also sorted using their putative origin (*A. viridis*, *Symbiodinium *sp or associated flora). For *N. vectensis*, 51% of the 27,273 predicted proteins were assigned GO terms.

Finally, this set of UniSeqs was annotated using similarity searches in both nucleotide and protein databases, as well as domain searches. Gene ontology terms were then assigned automatically using customized scripts based on InterProScan search results. We also submitted *N. vectensis *predicted proteins to the same InterProScan analysis for a comparative approach. To homogenize annotation level we only kept the root domain, using the hierarchical domain organization available from EBI. InterPro protein domains were identified in 40% of proteins. Based on similarity searches (Figure 3), we assigned putative taxa (*A. viridis*, *Symbiodinium *or associated flora) to each unique sequence. Figure 7 represents the distribution of assigned gene ontology terms. Comparison of domain occurrence in *A. viridis *with that in *N. vectensis *demonstrated that our library is highly representative of the Actinaria transcriptome. We also identified increased abundance of a number of molecular functions in *A. viridis *compared with *N. vectensis*: translation regulator activity (0.46 vs 0.23%), oxidoreductase activity (6.86 vs 4.98%), antioxidant activity (0.07 vs 0.03%), and structural molecule activity (3.24 vs 1.28%). It is noteworthy that some of these molecular functions are involved in symbiotic interactions with zooxanthellae, such as protein binding [24], nutrient transport and antioxidant activity [8]. For example, *A. viridis *counters the oxidative stress produced by the photosynthetic activity of its symbionts by using a great diversity of antioxidant defences [38]. Some biological processes were also increased in the *A. viridis *dataset compared with *N. vectensis*: secretion (0.81 vs 0.36%) and catabolism (0.65 vs 0.26%). The cytoplasmic components were also found to be more highly represented in *A. viridis *compared with *N. vectensis *(20.42 vs 9.59%).

All these differences between the present *A. viridis *dataset and the *N. vectensis *genome may reflect the crucial role of trophic exchanges between the sea anemone and its dinoflagellate symbionts, as well as specific host adaptations.

## Conclusion

This large EST collection has provided high quality data on all aspects of a temperate symbiotic cnidarian, particularly with regard to coding sequences and regulation features. For example, we identified many novel repeated elements (RE) in 3'UTRs, suggesting an invasion of most animal sequences by some specific RE families. It will be interesting to further investigate their potential biological role, particularly on gene regulation. Phylogenetic origin and functional classification of the holobiont sequences allowed the identification of several symbiotic candidate genes. These data are now being used to develop a dedicated microarray that will provide a valuable resource for future studies on symbiotic interactions in *A. viridis*. Furthermore, these data also have shown the importance of *Symbiodinium *symbionts as well as the associated flora of the three major prokaryotic species. Some sequences will be further analysed from the new perspective of gene transfer between host and symbiont. The relatively low abundance of sequences from *Symbiodinium *was attributed to experimental bias; a new ongoing sequencing project should fill this gap. Finally, these data from a temperate zone cnidarian provide novel molecular insights that will complement those obtained from tropical anthozoans. This dataset is valuable resource that will be of great help for comparative genomics and evolutionary studies.

## Methods

### Sampling and cDNA library construction

To maximize the diversity of genes expressed in the symbiotic association under both normal and stress conditions, sea anemones were subjected to different controlled stress conditions before RNA extraction and cDNA library construction.

Specimens of the Mediterranean sea anemone, *Anemonia viridis *(Forskål, 1775), were collected close to Villefranche-sur-mer (France). During an initial acclimatation period of at least 4 weeks, animals were maintained in tanks of running seawater at 17 ± 1°C with a light intensity of 250 μmol quanta m^-2^s^-1 ^(overhead metal halide lamps Philips HQI TS 400W), on a 12 h light/12 h dark cycle (starting at 8 am). Animals were fed once a week. After the acclimatation period, animals were treated and sampled (five tentacles from each specimen, four specimens from each experiment except for the hyperoxia stress) as follows:

• Light/dark cycle: sampling was performed at different times of the day (10 am and 7 pm) and night (7 am and 10 pm).

• Thermal stress: sea water was heated from 17°C to 24°C over 2·hrs and maintained at this maximal temperature for 5·days. Five *A. viridis *tentacles were sampled after 1, 2 and 5 days of continuous thermal stress.

• Hyperoxia condition: three specimens of *A. viridis *were subjected to 10·h of 100% O_2 _at 17.0 ± 1°C under a constant irradiance of 250·μmol·m^-2^·s^-1^. Oxygen saturation of the medium was achieved by bubbling pure O_2 _through seawater and was monitored using a gas analyser (Radiometer Copenhagen ABL 30; Copenhagen, Denmark).

• Bleached specimens, resulting from symbiosis disruption, and maintained bleached over several years (at 17°C in dark conditions) were also sampled.

The mRNAs were then extracted using Trizol Reagent (Invitrogen), as described in Richier et al. [39], and equal amounts of mRNA from the different conditions described above were combined before cDNA library construction. *A. viridis *cDNA library was generated at the RZPD (Deutsches Ressourcenzentrum für Genomforschung GmbH, Berlin, Germany) by oligodT, random priming and directionally cloning into pSPORT1. From this library, 50,304 clones were picked, replicated and subsequently sequenced at the Genoscope (French national sequencing centre). High-throughput sequencing of 5'end was performed using a Big-Dye terminator cycle sequencing kit and M13 reverse primer on an ABI-3730 Genetic Analyzer (Applied Biosystems) following the manufacturer's protocol (Genoscope, Evry, France). 41,247 chromatograms were thus generated and further analyzed.

### EST processing and analysis

EST sequences were processed using SURF analysis pipeline tools (SURF: SeqUence Repository and Feature detection, developed by the SIGENAE team, Dehais Patrice and Eddie Iannucelli, INRA, Toulouse). Basically, SURF provided an integrated solution, from chromatogram data storage to cloned insert detection, by integrating several dedicated bioinformatic software programs (sequence base calling, vector detection, etc.) in order to produce relevant nucleotide sequences according to base quality and feature detection. The chromatogram files were exported to PHRED for base calling [40,41]. Cloned insert detection was made according to different detected features (vector, adaptator, poly(A) or poly(T) tails and repeat) and their respectively positions, using third party programs (*Crossmatch *and *RepeatMasker*). Only inserts with more than 100 bp, with a Phred score >20, and not belonging to a low complexity area were exported into a fasta format with its corresponding quality file. Additional extremity trimming was made using the "trimseq" command (EMBOSS package). Low complexity regions and repeats were masked using the RepeatMasker program [42]. For this purpose, two different libraries were used: the RepeatMaskerLib (RepBase Update of 2007.09.24, [43]) and a custom library of *A. viridis*. This custom library was made both by using CENSOR [44] to retrieve publicly available repeats, and by running a BlastN of all ESTs against themselves to identify the most abundant repeat regions.

High quality ESTs (39,939) were then assembled into contigs using the TIGR-TGICL tool [32].

### Repeated elements analysis

Putative transposable elements (46 sequences) were first identified based on homology search after BlastX analysis against UniProt KB (E-value of < 1.10^-20^). An additional local BlastN search was performed using the EST dataset both as query sequence file and target database. Repeated motif sequences, i.e. repeats occurring more than twice from non overlapping ESTs, were selected as a first screen repeats dataset. These were used, together with the Repbase repeats library, to mask our EST sequences before clustering and assembling (TGICL). The same first screen dataset was then BlastN compared with the assembled database, and repeated motif sequences occurring on more than two different UniSeqs (E-value of < 1.10^-20^) were considered as *A. viridis *repeat sequences.

### Gene Ontology annotation

Gene functions were automatically assigned to 39% of the predicted proteins (5,652 UniSeqs). This assignment was based on the identification of InterPro (IPR) domains [45] using InterproScan [46] and the following command line: iprscan -cli -i unisequences.fa -o unisequences.ipr.raw -seqtype n -goterms -iprlookup -format raw. For comparative analysis of IPR domains found in the *A. viridis *dataset, we also ran the program InterproScan on the predicted *N. vectensis *proteome dataset. To homogenize the granularity level of annotation between organisms for each non-overlapping set of domains found, we only kept the root domain. We used the hierarchical organization of domains proposed in the "Parent-Child" description available on the EBI public ftp server . For example, all CYP proteins which have a P450 domain ([InterPro:IPR002949], [InterPro: IPR002397], [InterPro: IPR008070]) were counted at their root domain (IPR001128).

### Comparative genomic analysis

All contig and singleton sequences were compared with several databases, using Blast: the *Nematostella vectensis *draft genome (Predicted proteins, ), SwissProt (2008.03), TrEMBL (2008.03) and other ESTs from symbiotic cnidarian species (*Acropora millepora*, *Acropora palmata*, *Aiptasia pallida*, *Montastrea faveolata*) or from non symbiotic species (*Metridium senile*).

To confirm the origin of some selected genes, amplifications were performed on 10 ng of genomic DNA from *A. viridis *epidermal cells (non-symbiotic cells, animal origin [39]), cultured *Symbiodinium *cells extracted from *A. viridis *tentacles (non symbiotic cells, symbiont origin), and whole tentacle extracts (symbiotic cells, both animal and symbionts). Primers designed in the experiment are presented in Additional file 6 and were used in 40-cycle PCR reactions. *Elongation factor 1 alpha *and *Elongation factor 2 *were used as positive controls for nuclear-encoded genes from *A. viridis *and *Symbiodinium *spp, respectively, while *psbA *(photosystem II protein D1) was used as a positive control for chloroplast-encoded *Symbiodinium *spp genes. Sequence alignment was done using Multalin [47]. Signal peptide prediction was performed using SignalP [48]. Phylogenetic analyses were done using both MEGA 4.0 [49] and PHYML [50] software.

### Accession numbers

The ESTs generated in this study were submitted to dbEST ([GenBank:FK719875–FK759813]). Accession numbers of sequences used in MERP alignment: Am_MERP, *Acropora millepora*, [GenBank:EZ013381.1]; Mm_MERP, *Mus musculus*, [REFSEQ:NP_598826]; Hs_MERP, *Homo sapiens*, [REFSEQ:NP_060019]; Ca_MERP1, *Carassius auratus*, [GenBank: X14134.1]; Ca_MERP2, *Carassius auratus*, [GenBank: J04986.1].

## Authors' contributions

CS and PF contributed to the experimental design and sampling. CS oversaw the cDNA library construction and sequencing. CS, PG and ED participated in the assembly and annotation, performed comparative studies and drafted the manuscript. ED did the database work. PF and DA advised on all aspects of the work, with major contributions on sea anemone biology and writing. All authors read and approved the final manuscript.

## Supplementary Material

Additional file 1 ***Anemonia viridis *body organisation**. Body plan organisation of a symbiotic sea anemone (epiderm, mesoglea, gastroderm), and localisation of symbionts (zooxanthellae) within gastrodermal cells. Click here for file

Additional file 2 **Phylogenetic relationships among cnidaria**. The tree shows the relative position of the selected species cited in this study, according to M. Daly and N. Knowlton. Symbiotic species hosting zooxanthellae are highlighted (*). Click here for file

Additional file 3 **Summary of the EST analysis**. The table provides statistics on sequencing, assembling and analysis of *A. viridis *ESTs. Click here for file

Additional file 4 **Distribution of ESTs among contigs**. The distribution of the number of ESTs per contig presented as a histogram, with results grouped in classes of abundance (2–5, 6–10, 11–15, etc). Click here for file

Additional file 5 **Phylogenetic trees of Amine-oxidase sequences**. Three different methods of reconstruction have been used, Neighbor-Joining (**A**), Maximum Parsimony (**B**), and Maximum Likehood (**C**). The *A. viridis *gene has been compared to sequences from the stony coral *A. millepora *(GenBank: DY579173), the rhabditidae *Caenorhabditis elegans *(NCBI Reference Sequence: NM_059688.1) and *Caenorhabditis briggsae *(NCBI Reference Sequence: XM_001899776.1), the proteobacteria *Plesiocystis pacifica *(NCBI Reference Sequence: ZP_01909563.1) and *Beggiatoa *sp (NCBI Reference Sequence: ZP_02000236.1), and the cyanobacteria *Crocosphaera watsonii *(NCBI Reference Sequence: ZP_00514584.1) Click here for file

Additional file 6 **Primer sequences**. The Table shows the sequence of the primers used to amplify the selected metazoan genes from *A. viridis *epidermal genomic DNA. Click here for file
