# Supplementary figures and images for: Comprehensive EST analysis of the symbiotic sea anemone, Anemonia viridis
Source: BMC Genomics. 2009 Jul 23;10:333. doi: 10.1186/1471-2164-10-333 (PMC2727540; doi:10.1186/1471-2164-10-333)

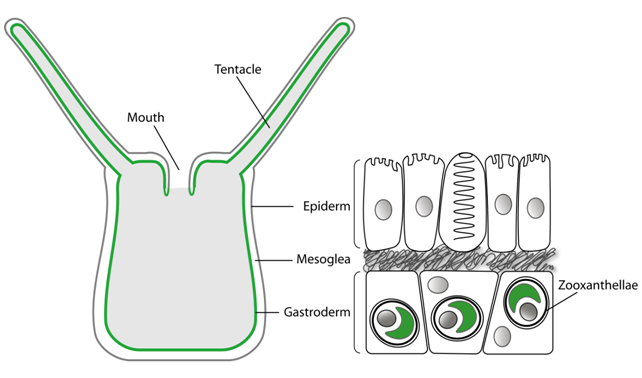

Supplement: Additional file 1 — Anemonia viridis body organisation. Body plan organisation of a symbiotic sea anemone (epiderm, mesoglea, gastroderm), and localisation of symbionts (zooxanthellae) within gastrodermal cells. [file 1471-2164-10-333-S1.png]

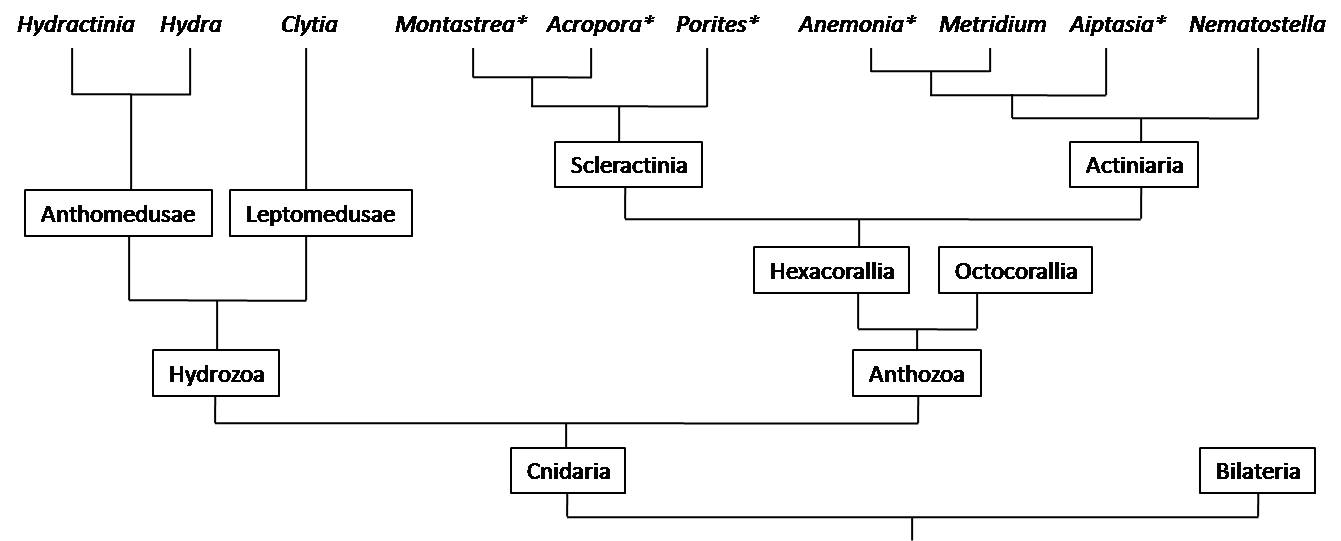

Supplement: Additional file 2 — Phylogenetic relationships among cnidaria. The tree shows the relative position of the selected species cited in this study, according to M. Daly and N. Knowlton. Symbiotic species hosting zooxanthellae are highlighted (*). [file 1471-2164-10-333-S2.jpeg]

## Slide 1
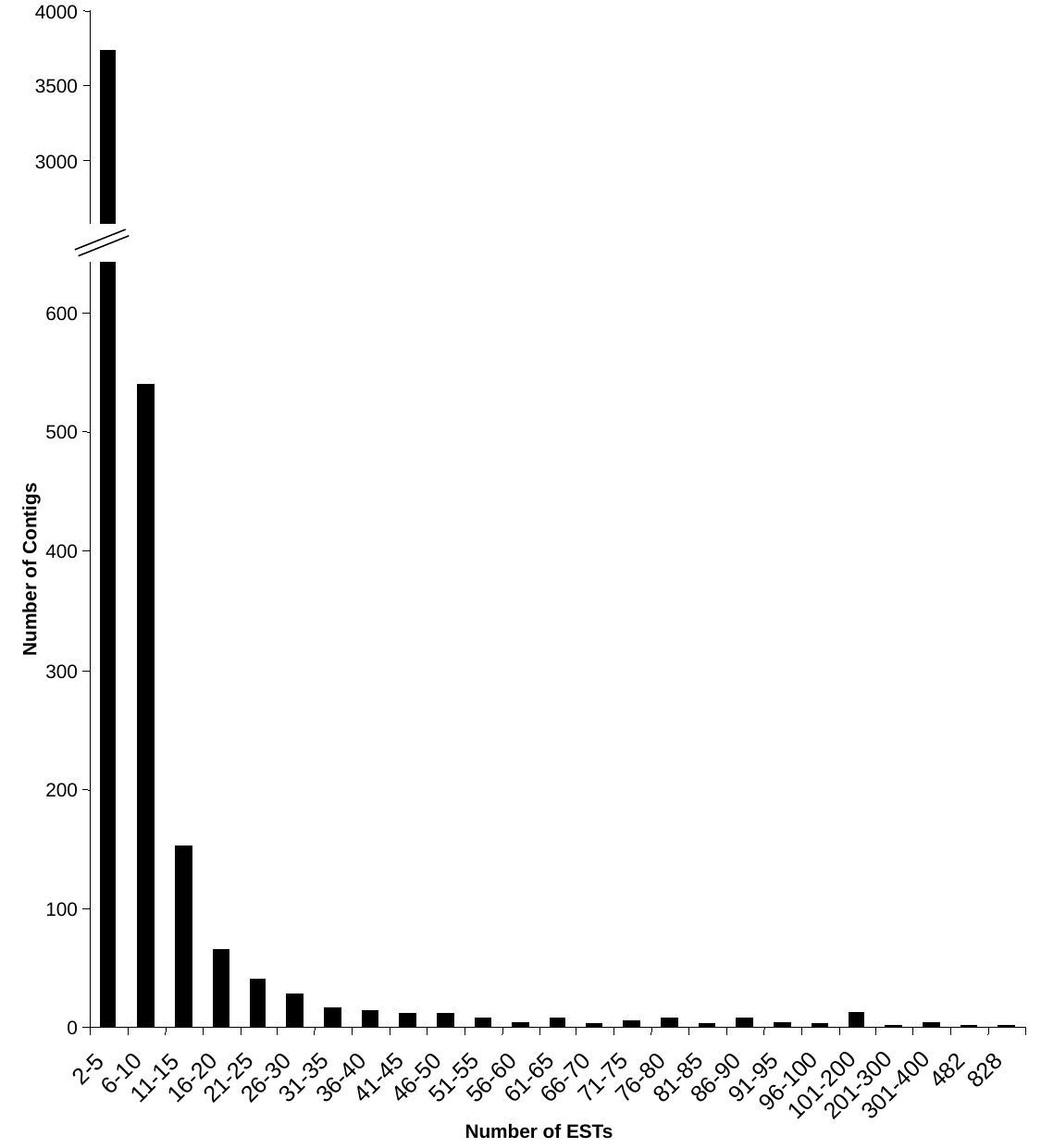

4000
3500
3000
600
500
400
300
200
100
0
2-5
482
828
6-10
66-70
11-15
16-20
21-25
26-30
31-35
36-40
41-45
46-50
51-55
56-60
61-65
71-75
76-80
81-85
86-90
91-95
96-100
101-200
201-300
301-400
Number of Contigs
Number of ESTs

Supplement: Additional file 4 — Distribution of ESTs among contigs. The distribution of the number of ESTs per contig presented as a histogram, with results grouped in classes of abundance (2–5, 6–10, 11–15, etc). [file 1471-2164-10-333-S4.ppt]
