# Supplementary material for: Comprehensive EST analysis of the symbiotic sea anemone, Anemonia viridis
Source: BMC Genomics. 2009 Jul 23;10:333. doi: 10.1186/1471-2164-10-333 (PMC2727540; doi:10.1186/1471-2164-10-333)
Supplement: Additional file 3 — Summary of the EST analysis. The table provides statistics on sequencing, assembling and analysis of A. viridis ESTs. [file 1471-2164-10-333-S3.doc]

| **Summary of the EST analysis** | |
| --- | --- |
| EST project statistics | |
| Total number of clones sequenced | 50304 |
| Number of chromatograms | 41247 |
| Number of high-quality ESTs | 39939 |
| Average length of trimmed ESTs (bp) | 626 |
| Number of unique sequences | 14504 |
| Number of contigs | 4652 |
| Number of ESTs in contigs | 30087 |
| Average assembled sequence length (bp) | 895 |
| Max. assembled sequence length (bp) | 3887 |
| Number of singletons | 9852 |
| Unigene annotations | |
| % blastx hits (UniProt 2008.03) | 43 |
| % blastx hits (SwissProt 2008.03) | 31 |
| % identification of IPR domains (InterProScan) | 39 |
| % GO terms assigned (InterProScan) | 29 |
