# Supplementary material for: Comprehensive EST analysis of the symbiotic sea anemone, Anemonia viridis
Source: BMC Genomics. 2009 Jul 23;10:333. doi: 10.1186/1471-2164-10-333 (PMC2727540; doi:10.1186/1471-2164-10-333)
Supplement: Additional file 5 — Phylogenetic trees of Amine-oxidase sequences. Three different methods of reconstruction have been used, Neighbor-Joining (A), Maximum Parsimony (B), and Maximum Likehood (C). The A. viridis gene has been compared to sequences from the stony coral A. millepora (GenBank: DY579173), the rhabditidae Caenorhabditis elegans (NCBI Reference Sequence: NM_059688.1) and Caenorhabditis briggsae (NCBI Reference Sequence: XM_001899776.1), the proteobacteria Plesiocystis pacifica (NCBI Reference Sequence: ZP_01909563.1) and Beggiatoa sp (NCBI Reference Sequence: ZP_02000236.1), and the cyanobacteria Crocosphaera watsonii (NCBI Reference Sequence: ZP_00514584.1) [file 1471-2164-10-333-S5.ppt]

## Slide 1
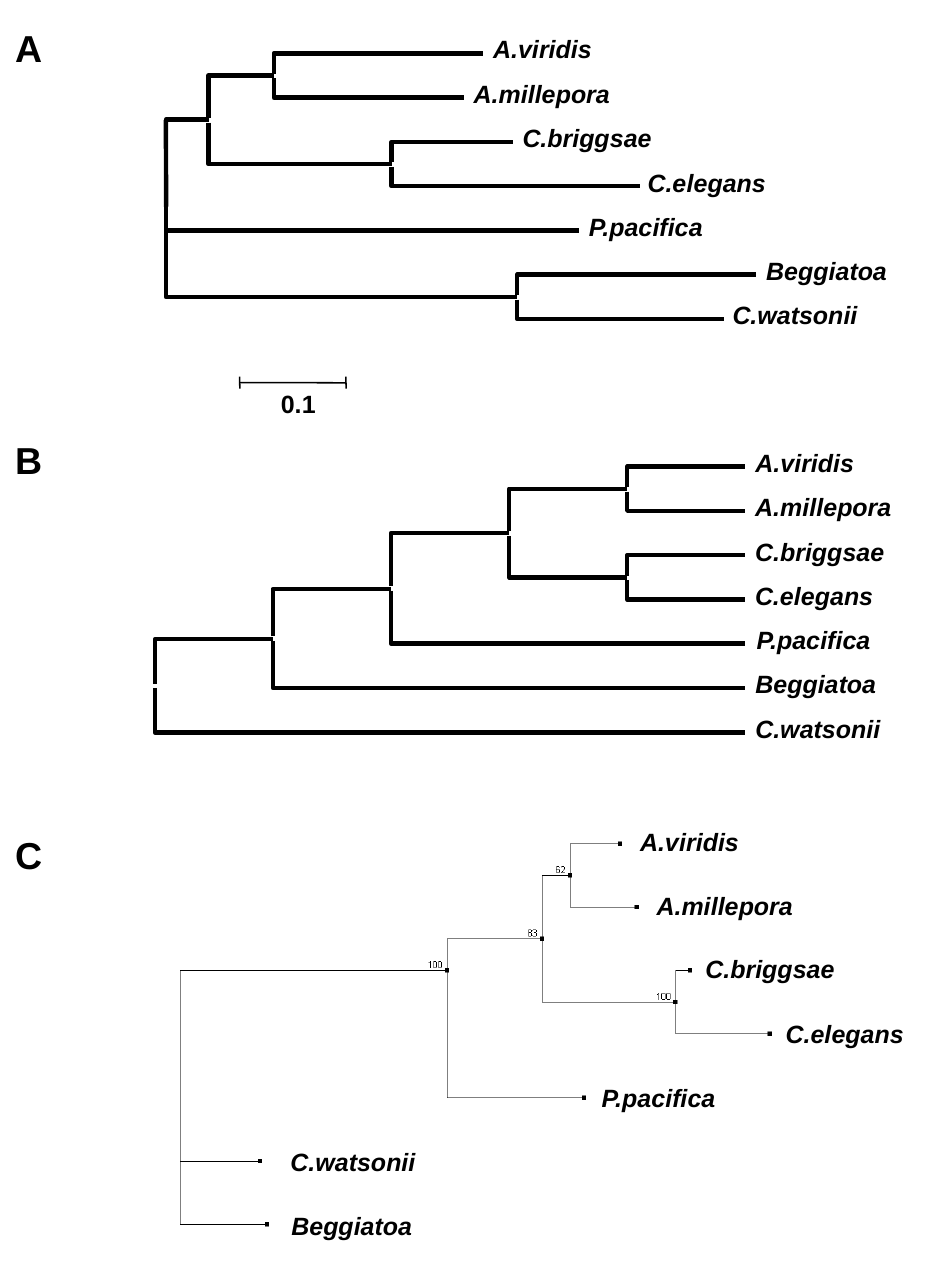

A
 A.viridis
 A.millepora
 C.briggsae
 C.elegans
 P.pacifica
 Beggiatoa
 C.watsonii
0.1
B
 A.viridis
 A.millepora
 C.briggsae
 C.elegans
 P.pacifica
 Beggiatoa
 C.watsonii
C
 A.viridis
 A.millepora
 C.briggsae
 C.elegans
 P.pacifica
 C.watsonii
 Beggiatoa
