# Supplementary material for: Comprehensive EST analysis of the symbiotic sea anemone, Anemonia viridis
Source: BMC Genomics. 2009 Jul 23;10:333. doi: 10.1186/1471-2164-10-333 (PMC2727540; doi:10.1186/1471-2164-10-333)
Supplement: Additional file 6 — Primer sequences. The Table shows the sequence of the primers used to amplify the selected metazoan genes from A. viridis epidermal genomic DNA. [file 1471-2164-10-333-S6.doc]

| **Primer sequences** | | | | |
| --- | --- | --- | --- | --- |
| Gene ID | Putative origin | Gene name | Forward primer (5'-3') | Reverse primer (5'-3') |
| CL27Contig1 | *A. viridis* | Elongation factor 1 alpha | GCCCACATTGCCTGTAAGTT | CCTTGGAGGGTTGAAGAGTG |
| CL51Contig1 | *A. viridis* | MERP-1 | ACCGTGACAGCAGAGGGTTG | GCTTCTGGGAGGCACGAATC |
| av02083n14r1.1 | *A. viridis* | Hephaestin-like (ferroxidase) | GGATGCAGGAATGGAAACCA | GGTTGCAGTCATCACCACCA |
| av02105j16r1.1 | *A. viridis* | Amine oxidase-like | TTTCCCAAGATACGGCCACA | GGCGACTGGTTGGAGATCCT |
| CL2879Contig1 | *Symbiodinium* | Elongation factor 2 | GCATGTGGATCACGGCAAGT | CACACCTCGCTCCTGCTCAT |
| av02090k02r1.1 | *Symbiodinium* | photosystem II protein D1 | GTAATCCCAAGCTCCAATGCAA | ATCCAGCAAGCCACACCAAG |
